# Insights into HKUST-1 Metal-Organic Framework’s Morphology and Physicochemical Properties Induced by Changing the Copper(II) Salt Precursors

**DOI:** 10.3390/ma18030676

**Published:** 2025-02-03

**Authors:** Joanna Klęba, Kun Zheng, Dorota Duraczyńska, Mateusz Marzec, Monika Fedyna, Jakub Mokrzycki

**Affiliations:** 1 Faculty of Energy and Fuels, AGH University of Krakow, Mickiewicza 30 Av., 30-059 Krakow, Polandzheng@agh.edu.pl (K.Z.); 2 Jerzy Haber Institute of Catalysis and Surface Chemistry, Polish Academy of Sciences, Niezapominajek 8, 30-239 Krakow, Poland; dorota.duraczynska@ikifp.edu.pl; 3 Academic Centre for Materials and Nanotechnology, AGH University of Krakow, Mickiewicza 30 Av., 30-059 Krakow, Poland; marzecm@agh.edu.pl; 4 Faculty of Chemistry, Jagiellonian University, Gronostajowa 2, 30-387 Krakow, Poland; monika.fedyna@uj.edu.pl

**Keywords:** HKUST-1, metal-organic framework, solvothermal synthesis, DMF-free synthesis

## Abstract

The HKUST-1 metal-organic framework was synthesized using four different copper(II) salt precursors, namely copper nitrate, copper sulphate, copper acetate, and copper chloride, via the solvothermal method with no mixing. Syntheses were conducted without using the N,N-dimethylformamide to allow for a greener synthesis of MOFs. The selected physicochemical properties of the obtained metal-organic frameworks were determined. The yield of the obtained products changed in the order acetate>nitrate>sulfate, while no product was obtained in the synthesis with copper(II) chloride. The obtained materials were characterized by means of XRD, nitrogen adsorption–desorption at −196 °C, FTIR, XPS, TGA, SEM, and DLS. The morphology of crystallites and their physicochemical properties were significantly affected when different copper(II) salt precursors were used. The comparison of the obtained results with already published works allows for the correlation of the synthesis parameters like synthesis temperature, time, mixing, and copper(II) salt precursor used on selected properties of the final product.

## 1. Introduction

In recent years, design and research on novel materials has gained major attention. A widely known group of crystalline materials known as MOFs—metal-organic frameworks—which were developed in the late 1990s, have brought a new insight into the synthesis of designable materials [1]. MOFs are a combination of two opposite components: inorganic metal clusters and organic linkers. Owing to the broad diversity of their selection—especially organic liners—there are at present over 123,000 reported MOF structures obtained at laboratory scale and over 500,000 structures that are possible to be obtained according to mathematical simulations.

Owing to unique surface properties, e.g., high specific surface area (>1000 m^2^ g^−1^), regular and designable porous structure, and adjustable surface active groups introduced at the synthesis stage or during post-synthesis procedures, MOFs have become important materials in chemical engineering. Impressively, the highest reported specific surface area for crystalline porous materials, 7800 m^2^ g^−1^, was obtained for MOF DUT-60 in 2018 [2]. MOFs’ most recent applications include gas separation and storage [3,4,5], heterogeneous catalysis [6,7,8], the adsorption of both organic and inorganic pollutants from aqueous media [9,10], photocatalysis [11,12,13], and drug delivery platforms [14,15,16]. However, their relatively low thermal stability limits their application in processes where elevated temperatures >250 °C are required [17].

Among numerous MOF structures, HKUST-1 (Hong Kong University of Science and Technology-1), also known as MOF-199, has gained much attention due to the ease of its synthesis [18]. HKUST-1 possesses a 3-dimensional porous structure composed of copper ion clusters coordinated with 1,3,5-tribenzenecarboxylic acid (trimesic acid). The specific surface area is usually >1000 m^2^ g^−1^, while the size of the HKUST-1 pore window is about 0.9 nm [19].

The major factor impeding the properties of the final product is the synthesis procedure, which can be conducted in various modes: solvothermal synthesis (with or without mixing or with an additional energy source, either microwaves or ultrasounds) [18,20], mechanochemical synthesis [21,22], and electrochemical synthesis [23,24]. Among the abovementioned methods, solvothermal synthesis is the most widely applied [25]. However, it requires the removal of the solvent from the pores to allow for surface activation and improvement in the specific surface area.

In the work by Zhang et al. [26], the authors claimed that, with the synthesis of HKUST-1 using copper(II) nitrate and ethanol as solvent, phase purity was not obtained and additional phases occurred in the PXRD diffractogram, despite the fact that the crystals exhibited HKUST-1-like morphology (octahedral crystallites). The effect of various salt precursors on the physicochemical properties of obtained HKUST-1 MOFs was investigated by Liu et al. [27]. The yield of the final product varied when different salt precursors were used; however, the syntheses were conducted in the presence of DMF (N,N-dimethylformamide) and ethanol as organic solvents (with or without the addition of triethylamine—TEA). Regardless of the salt precursor used, the phase composition was maintained; however, the specific surface area, pore size, and product yield were significantly changed. Moreover, recent studies imply that DMF, which is a widely applied volatile organic compound, may contribute to liver, lung, and testicular cancers [28]. DMF is metabolized in the liver by the microsomal cytochrome P4502E1 (CYP2E1); however, its negative impact on human health is gaining attention. For this reason, a trend towards removing its application in organic syntheses (including MOFs) is in line with the principles of green chemistry [29].

The aim of the present study was to demonstrate the physicochemical properties of the HKUST-1 MOFs obtained using four different copper(II) salt precursors in a DMF-free solvothermal synthesis procedure. The main outcome of this work was to show how the phase composition, specific surface area, pore volume, thermal stability, and surface morphology of the final product can be affected by changing the copper(II) salt precursors. The study is a continuation of our former research [9], in which a similar synthesis procedure was employed, although no mixing was then involved. The results obtained were correlated with already published works, where various synthesis parameters were also evaluated. The knowledge gap in the broad comparison of various copper(II) salt precursors’ effects on the properties of HKUST-1 MOFs was filled.

## 2. Materials and Methods

### 2.1. HKUST-1 Syntheses

**Figure 1:**
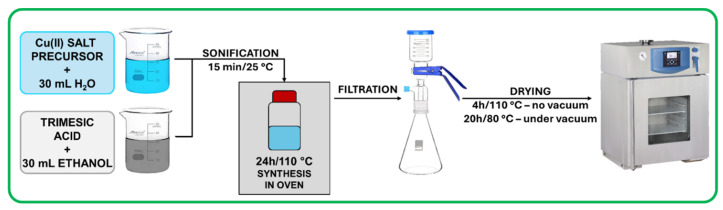
HKUST-1 synthesis scheme.

HKUST-1 was synthesized using a conventional solvothermal method described in the work of Chen et al. [30]. According to this method, two separate solutions were prepared containing 9 mmol of copper(II) salt precursor in 30 mL of deionized water and 5 mmol of trimesic acid (Sigma Aldrich, Burlington, MA, USA) in 30 mL of ethanol (96 % purity, Stanlab, Lublin, Poland), respectively. The selected copper(II) salt precursors were Cu(NO_3_)_2_·3H_2_O (Aktyn, Suchy Las, Poland), (CH_3_COOH)_2_Cu·H_2_O (Fluka, Buchs, Switzerland), CuCl_2_·2H_2_O (Sigma Aldrich, Burlington, MA, USA), and CuSO_4_·5H_2_O (Sigma Aldrich, Burlington, MA, USA). Solutions were mixed together in a 100 mL bottle and placed in an ultrasound bath (ULTRA-080S, Sonicco, Warsaw, Poland) for 15 min at 25 °C to initiate the nucleation. Next, samples were placed in an oven for 24 h at 110 °C to finalize the synthesis. Samples were then filtrated using a nylon membrane filter of 0.45 μm pore size, washed several times with water, and dried in oven for 4 h at 110 °C followed by drying for 20 h at 80 °C under vacuum. Samples were denoted a-HKUST-1, c-HKUST-1, n-HKUST-1, and s-HKUST-1, where the letter corresponds to the copper(II) salt precursor used: acetate, chloride, nitrate, and sulfate, respectively. A schematic illustration of the synthesis procedure is presented in Figure 1.

### 2.2. Materials Characterization

#### 2.2.1. X-Ray Diffraction (XRD)

The phase composition of the investigated MOFs was determined using a PANalytical Empyrean diffractometer (Malvern Panalytical, Malvern, UK). The source of radiation was CuKα λ = 1.5406 Å, and the 2θ range was 5–45° with a 0.013° step.

#### 2.2.2. Nitrogen Adsorption-Desorption at −196 °C

The adsorption–desorption isotherms of nitrogen were collected at −196 °C using an ASAP 2020 instrument (Micromeritics, Norcross, GA, USA). Prior to the measurement, samples were degassed at 150 °C for 12 h. From the isotherms obtained, the specific surface area (S_BET_), total pore volume (V_t_)at p/p_0_ of approx. 0.90, and micropore volume (V_mic_), using the H-K method, of the investigated MOFs were calculated using the MicroActive *V* 4.06 software (Micromeritics, Norcross, GA, USA).

#### 2.2.3. Fourier-Transform Infrared Spectroscopy (FTIR)

IR spectra were collected at 1 cm^−1^ resolution using a spectrometer (Nicolet 6700, Thermoscientific, Madison, WI, USA) operating in Attenuated Total Reflection mode (ATR-FTIR).

#### 2.2.4. X-Ray Photoelectron Spectroscopy (XPS)

The analyses were carried out in a PHI VersaProbeII Scanning XPS system using monochromatic Al Kα (1486.6 eV) X-rays. The photoelectron take-off angle was 45°, and the pass energy in the analyzer was set to 117.50 eV for survey scans and 46.95 eV to obtain high-energy resolution spectra for the C 1s, O 1s, and Cu 2p regions. A dual beam charge compensation with 7 eV Ar^+^ ions and 1 eV electrons was used to maintain a constant sample surface potential regardless of the sample conductivity. All XPS spectra were charge-referenced to the unfunctionalized, saturated carbon (C-C) C 1s peak at 285.0 eV. The operating pressure in the analytical chamber was <3 · 10^−9^ mbar. The deconvolution of spectra was carried out using the PHI MultiPak software (v.9.9.3). Spectrum background was subtracted using the Shirley method.

#### 2.2.5. Thermogravimetric Analysis (TGA)

The samples’ mass loss at increasing temperature in an inert medium was measured using the Q5000IR apparatus (TA Instruments, New Castle, DE, USA). 30 mg of sample was weighed and heated from room temperature to 700 °C with a heating rate of 5 °C min^−1^ and a 100 mL min^−1^ flow of argon.

#### 2.2.6. Dynamic Light Scattering (DLS)

The particle size was measured by means of the dynamic light scattering method using a Mastersizer 3000 laser-diffraction analyzer (Malvern Panalytical, Malvern, UK).

#### 2.2.7. Scanning Electron Microscopy (SEM)

The imaging of the samples’ morphology was conducted using JEOL JSM 7500 F (JEOL, Tokyo, Japan). Samples were first coated with a thin (30 nm) layer of chromium using the K575X Turbo Sputter Coater (Quorum Emitech, South Stour Avenue, Ashford, UK).

## 3. Results and Discussion

The obtained product yields and pH of the synthesis are summarized in Table 1. It was evidenced that the precursors of the used copper(II) salts have a major impact on the yield of the final product. The yield of the HKUST-1 products changed in the following order (depending on the used copper(II) salt precursor): acetate > nitrate (1.8 times less) > sulfate (4.6 times less); in contrast, synthesis with copper(II) chloride yielded no product. A similar observation was confirmed in our latest work [9], where such a phenomenon was also observed. What is noteworthy is that the yield of a-HKUST-1, which was roughly 96.3%, might be a result of competition of the AcO^−^ ions with the organic linker BTC^3−^ ions during the crystallization process. It may also suggest the partial incorporation of these ions into the structure of the formed MOF [31,32]. The solution pH after synthesis had a rather negligible effect on the final product yield and was maintained in the acidic pH range in all cases (see Table 1).

**Figure 2:**
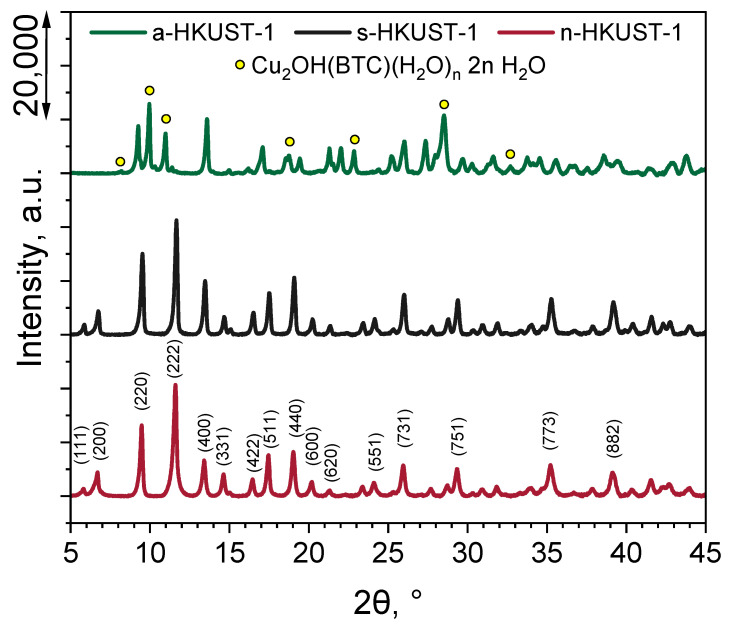
XRD pattern of the obtained MOFs.

The results of the crystallographic plane composition of the obtained HKUST-1 MOFs are presented in Figure 2. Regardless of the used copper(II) salt precursor, the HKUST-1 structure was obtained using the investigated procedure. According to the PDF-4+ powder diffraction database (JCPDS 00-064-0936) [33], the peak position was 2θ, and the corresponding identified planes were 5.80° (111), 6.70° (200), 9.48° (220), 11.66° (222), 13.41° (400), 14.62° (331), 16.46° (422), 17.46° (511), 19.02° (440), 20.21° (600), 21.32° (620), 24.12° (551), 25.98° (731), 29.32° (751), 35.25° (773), and 39.15° (882). However, in the case of a-HKUST-1 (Figure 2, green line), some additional planes were identified and were a result of the formation of a Cu_2_OH(BTC)(H_2_O)_n_·2n H_2_O phase, caused by the presence of acetate ions in the reaction medium and already reported by Crawford et al. [34] and in our recent work [9]. The peaks located at 2θ=8.10, 9.96, 18.74, 22.84, 28.49, and 32.70°, indicated by yellow dots in Figure 2, correlate well with the reports of Loera-Serna et al. [35] and Rodríguez-Esteban et al. [36] and confirm the presence of the Cu_2_OH(BTC)(H_2_O)_n_·2n H_2_O phase in the a-HKUST-1 sample.

**Figure 3:**
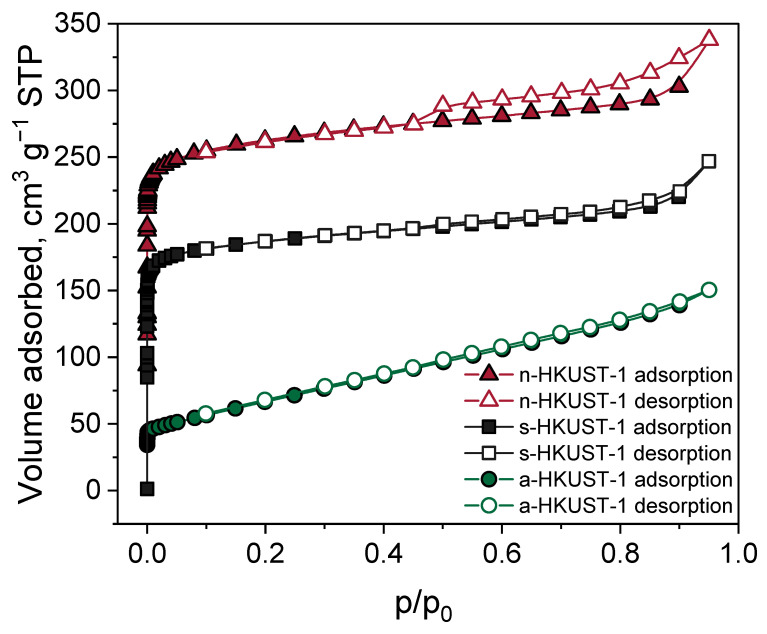
Nitrogen adsorption–desorption isotherms of investigated MOFs at −196 °C.

Nitrogen adsorption–desorption at −196 °C was employed to investigate the specific surface area (S_BET_) and characteristics of the pores. The results are summarized in Figure 3 and Table 2. The S_BET_ of the obtained MOFs changed in the order n-HKUST-1 (1153 m^2^ g^−1^*)* > s-HKUST-1 (937 m^2^ g^−1^) > a-HKUST-1 (553 m^2^ g^−1^). For all the investigated samples, the isotherm shape exhibited a hybrid type IA/II behavior according to the IUPAC classification, implying a microporous material with strong adsorbate–adsorbent interactions. At the same time, they possessed a type H3 or H4 hysteresis loop, which might be a result of aggregated crystals and/or occurring mesoporosity [37].

The sample n-HKUST-1 displayed a 43.3% share of micropores in total pore distribution (see Table 2). The highest number of micropores was found in s-HKUST-1 (59.5%), for which the hysteresis loop was less visible than for n-HKUST-1. In the case of a-HKUST-1, there was no hysteresis loop observed under relatively high pressure (p/p_0_), which indicates the least developed porous structure of the material among the investigated series, with a S_BET_ of 553 m^2^ g^−1^ and only 22.0% of micropores (see Table 2). The significant reduction in S_BET_ and V_micro_ (compared to the other materials) for the a-HKUST-1 sample can be a result of the formation of the Cu_2_OH(BTC)(H_2_O)_n_·2n H_2_O phase in the pores and/or on the surface of the HKUST-1 crystals.

**Figure 4:**
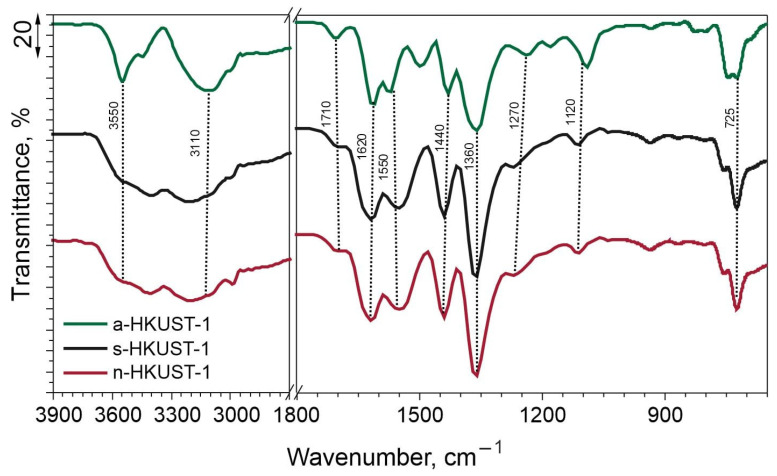
FTIR spectra of the obtained MOFs.

FTIR analysis was conducted to investigate the surface functional groups. The results are summarized in Figure 4. It can be clearly seen that the all the samples displayed a similar shape of characteristic bands in the examined wavenumber regions. Stretching bands originating from Cu-O were centered at ~725 cm^−1^. The presence of the FTIR peak at ∼725 cm^−1^ confirms the formation of a metal-linker bond between Cu and BTC [38]. At about 1090 cm^−1^, a band from Cu-O-C can be seen for a-HKUST-1, while for s-HKUST-1 and n-HKUST-1 it is slightly shifted to about 1110 cm^−1^. Bands at around 1620, 1440, 1360, and 1270 cm^−1^ were assigned to symmetric and asymmetric stretching vibrations of C=O and C-O vibrations from 1,3,5-benzenetricarboxylic acid units [39,40]. Moreover, the peak at about 1550 cm^−1^ was identified as C=C asymmetric vibrations of the aromatic ring. The peak originating from the BTC acid at 1710 cm^−1^ was also reported by Farzaneh et al. [41]. Peaks assigned to OH- groups were identified in the region >3000 cm^−1^ and confirm the presence of accompanying water within the material pores or loosely bonded to the structure of MOF, as seen for a-HKUST-1 (3550 cm^−1^) [42].

**Figure 5:**
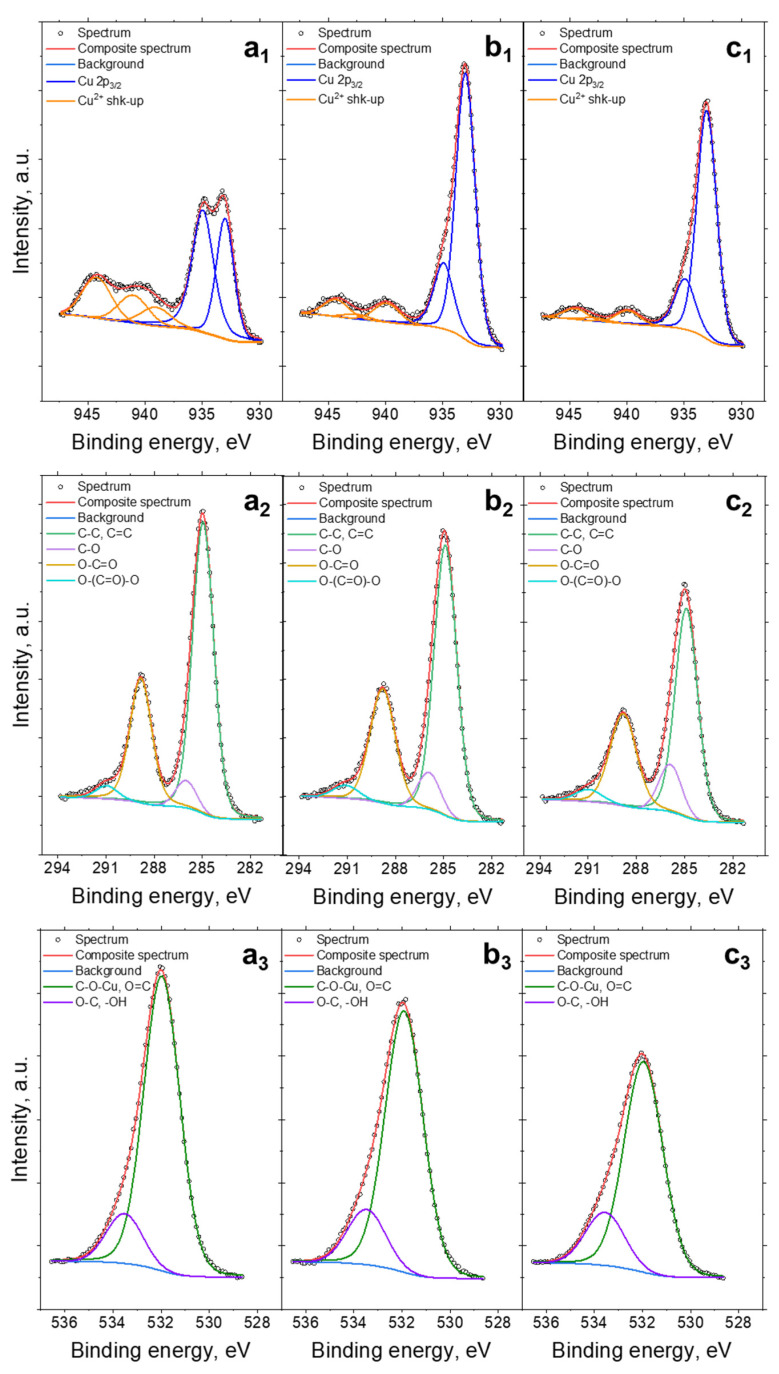
XPS spectra of Cu 2p (**a_1_**–**c_1_**), C 1s (**a_2_**–**c_2_**), and O 1s (**a_3_**–**c_3_**) for a-HKUST-1 (**a**), s-HKUST-1 (**b**), and n-HKUST-1 (**c**) samples.

X-ray photoelectron spectroscopy (XPS) was used to characterize the HKUST-1 samples and determine the chemical bond concentration on their surface. The fitted Cu 2p, C 1s and O 1s spectra are presented in Figure 5 and summarized in Table 3. The spectra collected at the Cu 2p_3/2_ region (see Figure 5a_1_–c_1_) were fitted with two components, the first line centered at 933.0 eV and the second at 935.0 eV. However, in the case of materials containing copper incorporated into their structure, it is difficult to assign a particular line to one Cu species in a univocal way, due to the reducibility of Cu^2+^ in the vacuum chamber during the XPS measurement [43,44]. This can make it difficult to delineate properly the Cu^2+^ and Cu^+^ species in the examined samples. However, the presence of shake-up structures (Cu satellites) found within the binding energy range of 940–945 eV confirms a high share of Cu^2+^ in the investigated HKUST-1 samples [43,44]. The share of Cu according to the XPS measurement was comparable for all the samples and varied from 10.4 to 10.6 at.% (Table 3). As Figure 5a_2_–c_2_ shows, the C 1s spectrum has four peaks, which are respectively matched to C–C (285.0 eV), C-O (286.0 eV), O-C=O (288.8 eV), and O-(C=O)-O (291.0 eV) [45]. The sum of the C atomic content was 54.6, 55.6, and 55.6% for a-HKUST-1, n-HKUST-1, and s-HKUST-1, respectively. The O 1s spectra were fitted with two components: a first line centered at 532.0 eV which mainly comes from metal oxides (O-Cu) and/or O=C type bonds and a second line found at 533.5 eV indicating O-C type bonds from organic material (see Figure 5a_3_–c_3_) [5,6]. The share of oxygen atomic content was the highest for a-HKUST-1 at 34.9%, implying the formation of a Cu_2_OH(BTC)(H_2_O)_n_·2n H_2_O phase. For n-HKUST-1 and s-HKUST-1 the share was comparable and as high as 33.9%.

**Figure 6:**
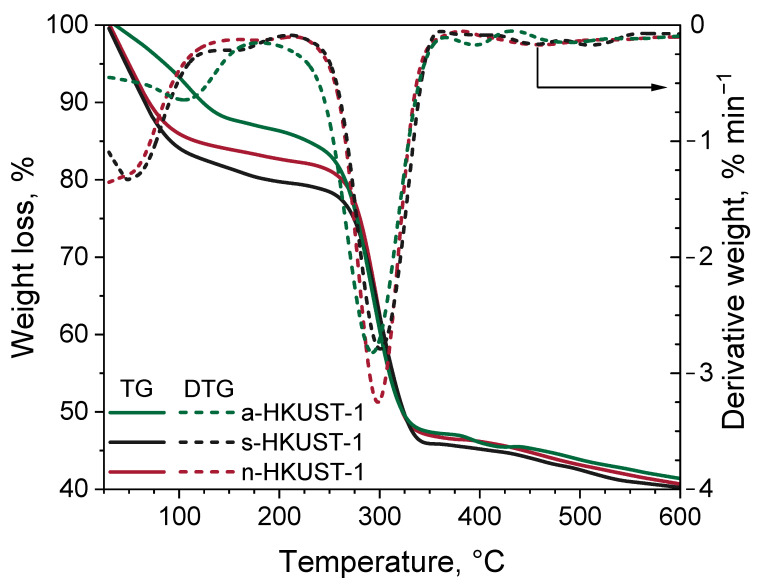
TG (solid lines, **left** y-axis) and DTG (dash lines, **right** y-axis) analysis of investigated MOFs.

The thermal stability of the obtained materials was investigated by means of TG. The results are presented in Figure 6. From the DTG curve it can be clearly seen that there were two main weight loss occurrences during sample heating. For n-HKUST-1 and s-HKUST-1 the first weight loss maximum occurred at about 50 °C and was related to the removal of ethanol from the pores of the material, while for a-HKUST-1 the first weight loss maximum occurred at about 106 °C and was related to loss of water probably from the Cu_2_OH(BTC)(H_2_O)_n_·2nH_2_O phase [34].

The second maximum (about 20% weight loss) observed for the s-HKUST-1 and n-HKUST-1 samples at about 100 °C was related to the release of physically adsorbed water molecules located in the pores of HKUST-1. The 20% weight loss in this temperature range is consistent with the observations of Ennis at al. [46]. On the other hand, a small loss in mass associated with water release from s-HKUST-1 and n-HKUST-1 was observed at about 160 °C; it may have been caused by the removal of water which remained in micropores until this temperature and might have been desorbed from the Cu paddle-wheel sites (chemisorbed water). The main weight loss was observed for all the materials at 300 °C and was related to the decomposition of the BTC organic linker [47]. Hence, the used salt precursor has no effect on the improvement of the thermal stability of the HKUST-1 structure, and the decomposition profile was comparable for all the samples in the temperature range from 300 to 600 °C.

**Figure 7:**
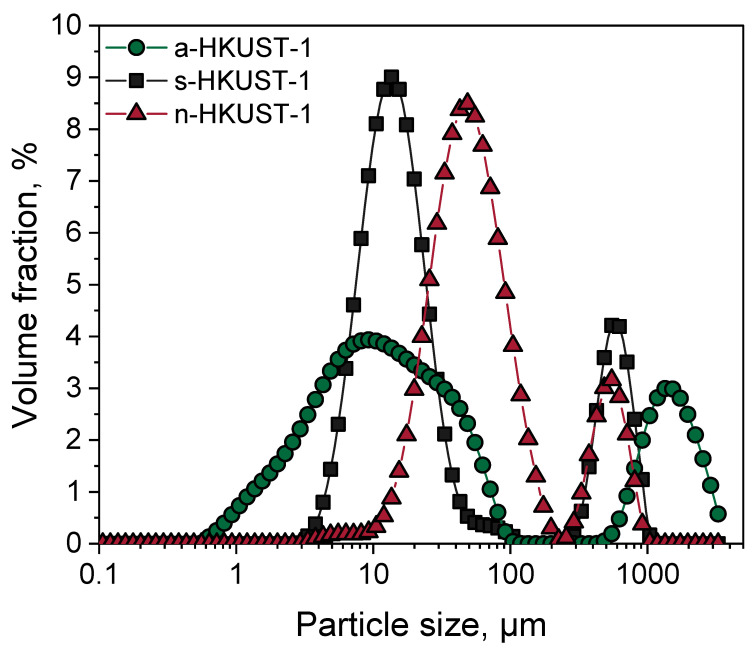
DLS analysis of the particle size of investigated MOFs.

The DLS particle size analysis results are presented in Figure 7. For all MOFs investigated, two particle size maxima were obtained: 12 and 550 μm for s-HKUST-1 and 48 and 550 μm for n-HKUST-1; for a-HKUST-1, meanwhile, a broad size distribution was obtained in the region from 1 to 100 μm (maximum at 10 μm and 40 μm), and a second one at about 1500 μm. Copper(II) sulfate favors the formation of smaller particles when compared to the acetate derived HKUST-1.

**Figure 8:**
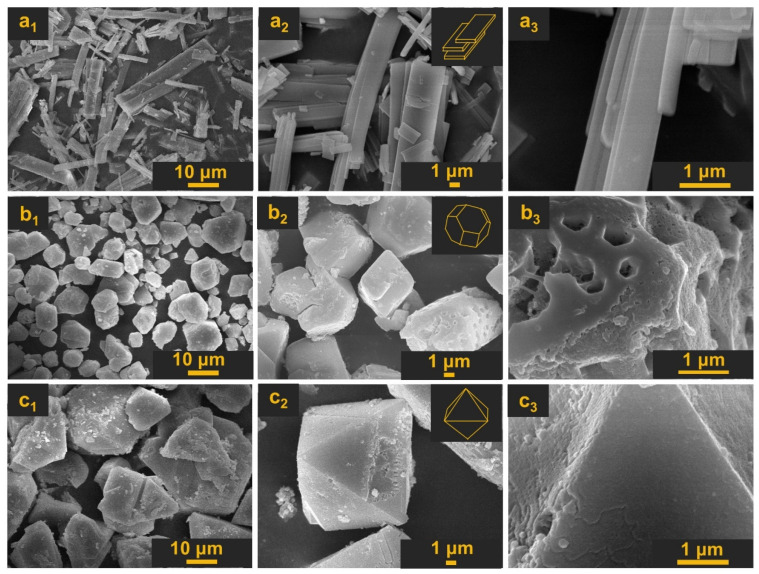
SEM images of investigated MOFs: a-HKUST-1 (**a_1_**–**a_3_**), s-HKUST-1 (**b_1_**–**b_3_**), and n-HKUST-1 (**c_1_**–**c_3_**), at magnifications of 1500 (1), 5000 (2), and 25000 (3).

SEM images were collected to investigate the surface morphology of the obtained MOFs. The results are summarized in Figure 8. The surface morphology significantly changed when various copper(II) salt precursors were used. In Figure 8a_1_–a_3_, it can be seen that the surface of HKUST-1 obtained from copper(II) acetate displays a board-like morphology of various sizes. The morphology is similar to the samples obtained in a procedure with mixing [9]. In the case of samples obtained from copper(II) sulfate (Figure 8b_1_–b_3_) and copper(II) nitrate (Figure 8c_1_–c_3_), the morphology was more uniform and bipyramid-like, and significantly bigger crystals are formed in the no-mixing procedure in comparison with the mixing procedure. The sizes of the obtained crystals correlate well with the DLS results, and the average crystal diameters were about 16 μm (s-HKUST-1) and 55 μm (n-HKUST-1).

## 4. Summary

The above described HKUST-1 MOFs DMF-free synthesis allowed us to compare the effect of four different copper(II) salt precursors on the surface properties and morphology of obtained crystals. In Table 4, a comparison of various synthesis parameters of HKUST-1 MOFs on their physicochemical properties with those seen in already published works are summarized. It can be seen that the most commonly used copper(II) salt precursor is copper(II) nitrate, whereas other salt precursors are seldom used. The temperature of the synthesis, its time, and the synthesis procedure selected appear to significantly impact the properties of the final product. A trend observed among the summarized works brings the conclusion that a synthesis time >3 h and a temperature ranging from about 80 to 110 °C allows one to obtain HKUST-1 crystals characterized by a relatively high specific surface area >1000 m^2^ g^−1^. The addition of DMF or small concentrations of acetic ions to the synthesis medium favors more regular crystal formation. However, the necessity of its removal from the pores of the formed MOF increases the costs of the final product. Moreover, the presence of acetic ions can lead to the occurrence of additional phases. The presence of water during the synthesis also plays a crucial role in the properties of the crystals, and a water:ethanol ratio of 1:1 was reported to be the optimal. The application of an extra source of energy like ultrasounds or microwaves leads to the faster nucleation of crystals. Furthermore, by the simple involvement of a mixing procedure, the specific surface area appears to be increased, while the formed crystals are relatively smaller (up to 5-fold) and more uniform, although the effect of mixing can vary depending on the used temperature and time.

Taking into account the changes in surface properties and morphology induced by synthesis parameters, the potential applications of HKUST-1 MOFs can be adapted. Greater specific surface area and bigger pore size distribution can contribute to an enhanced adsorption of organic dyes [48] or inorganic ions like Pb(II) [49], Ce(III) [50], and Cr(III) [9] from aqueous solutions. It should be noted, however, that the structure of HKUST-1 is reported to posses relatively low stability in water, which limits its adsorption applications.

A relatively low thermal stability <300 °C and the presence of organic linker make application in thermal processes impossible. HKUST-1 was, however, evaluated in a 4-nitrophenol to 4-aminophenol catalytic reduction [6] or CO oxidation [51]. It was clearly indicated that the occurrence of surface defects like missing ligands can accelerate CO oxidation by changing the reaction pathways and lowering the activation energy. Defects formed by changing the ligand can also favor the formation of hierarchical porosity, which can promote the application of HKUST-1 as membranes for CO_2_ and SO_2_ adsorption [52]. Hence, the study on various HKUST-1 geometries may provide a valuable background in the catalytic and adsorption application of MOFs.

**Table 4:** Comparison of HKUST-1 synthesis methods and selected physicochemical properties of obtained products.

Synthesis Conditions				Selected Properties				Ref.
Synthesis Method	Solvent	Copper(II) Precursor	Conditions	Surface Properties		Crystal Properties		
				S_BET_, m^2^ g^−1^	V_t_, cm^3^ g^−1^	Morphology	Average Size	
Solvothermal	DMF	Cu(NO_3_)_2_ 3H_2_O	T = 140 °C t = 5–30 min mixing involved	2.60–12.02	-	Spheric nanoparticles	200 nm	[18]
Ultrasound assisted solvothermal	DMF	Cu(NO_3_)_2_ 3H_2_O	T = 80 °C t = 24 h no mixing	977.00	0.387	Bipyramid	-	[53]
Solvothermal	DMF, ethanol	Cu(NO_3_)_2_ 3H_2_O	T = 25 °C t = 1 min mixing involved, ZnO as synthesis modifier	1489.00	0.660	Bipyramid	605 nm	[49]
Solvothermal	Water, ethanol	Cu(NO_3_)_2_ 2.5H_2_O	T = 25 °C t = 14–34 h mixing involved, acetic acid as modifier	-	-	Bipyramid—low acetic acid concentration Rod-like shape—elevated acetic acid concentration	~300 nm ~600 nm	[48]
Solvothermal	DMF	Cu(NO_3_)_2_ 3H_2_O	T = 125 °C t = 24 h	1617.00	0.767	Bipyramid	25,000–40,000 nm	[25]
	Water, ethanol		T = 125 °C t = 24 h no mixing	1648.00	0.816		5000–15,000 nm	
	DMF		T = 104 °C t = 30 min no mixing	-	-		5000–20,000 nm	
Solvothermal	Water	Cu_2_CO_3_(OH)_2_ H_2_O	T = 25 °C t = 0.3–24 h no mixing	10.00–130.00 (lower for longer time)	-	Rod-like shape	-	[54]
	Water:Ethanol (1:1)			900.00–1600.00 (the highest for 3 h)		Bipyramid	-	
	Ethanol			25.00–160.00 (lower for shorter time)		Undefined agglomerates	-	
Solvothermal	Water:Ethanol (1:1)	Cu(NO_3_)_2_ 3H_2_O	T = 110 °C t = 24 h Mixing involved	1453.00	0.671	Irregular	~10,000 nm	[9]
		CuCl_2_ 2H_2_O		*No product*				
		CuSO_4_ 5H_2_O		1790.00	0.688	Irregular	~10,000 nm	
		Cu(CH_3_COO)_2_ H_2_O		961.00	0.418	Rod-like shape	1000–100,000 nm	
Ultrasound assisted solvothermal	Water:Ethanol (1:1)	Cu(NO_3_)_2_ 3H_2_O	T = 110 °C t = 24 h Ultrasounds for 15 min at 25 °C, than no mixing	1153	0.469	Bipyramid	~50,000 nm	This study
		CuCl_2_ 2H_2_O		*No product*				
		CuSO_4_ 5H_2_O		937.00	0.341	Irregular	~10,000 nm	
		Cu(CH_3_COO)_2_ H_2_O		553.00	0.209	Rod-like shape	1000–100,000 nm	

## 5. Conclusions

From the experiments conducted it was clearly evidenced that the use of various copper(II) salt precursors significantly affects HKUST-1 properties. Not only the product yield, but also specific surface area, surface morphology and crystals sizes are sensitive to the synthesis environment conditions. Synthesis without the addition of DMF allows one to obtain an HKUST-1 phase sufficiently comparable to already published materials’ physicochemical properties. The occurrence of chloride ions blocs synthesis, and no product can be obtained in a modulator-free synthesis with copper(II) chloride used as salt precursor. When acetate ions are present in the synthesis environment, the nucleation is rapid and begins immediately at room temperature, yielding the highest amount of the final product. However, acetate ions can compete with BTC^3−^ ions and can be partially introduced into the MOF structure, which has a significant impact on the properties of the product and its phase purity. In fact, the presence of an additional phase of Cu_2_OH(BTC)(H_2_O)_n_·2n H_2_O was confirmed in the obtained product. Depending on the desired properties of the final product, copper(II) nitrate, copper(II) sulfate, and copper(II) acetate can be successfully used in the preparation of HKUST-1 MOF. By removing mixing from the synthesis procedure, the obtained crystals are more uniform and even fivefold bigger in comparison to their counterparts obtained with the mixing procedure. Their porosity and, consequently, their specific surface area are consequently lower.

## Figures and Tables

**Table 1:** Product yield and pH of the synthesis of HKUST-1 using various salt precursors.

Parameters	a-HKUST-1	c-HKUST-1	n-HKUST-1	s-HKUST-1
Product yield, %	96.3 ± 0.1	0	52.3 ± 0.2	21.2 ± 0.1
Synthesis pH, -	3.92 ± 0.12	2.31 ± 0.02	2.96 ± 0.08	2.06 ± 0.03

**Table 2:** Surface properties of investigated MOFs.

Parameters	a-HKUST-1	n-HKUST-1	s-HKUST-1
S_BET_, m^2^ g^−1^	553	1153	937
V_t_, cm^3^ g^−1^	0.209	0.469	0.341
V_micro_, cm^3^ g^−1^	0.046	0.203	0.244
V_micro_/V_t_, %	22.0	43.3	59.5

**Table 3:** Surface composition (atomic %) determined by fitting XPS spectra.

Element	C				O		Cu
Binding energy, eV	285.0	286.0	288.8	291.0	532.0	533.5	932.5–933.5
Groups/Oxidation state	C-C	C-O	O-C=O	O-(C=O)-O	C-O-Cu O=C	O-C	Cu^+/2+^
a-HKUST-1	34.1	3.0	15.9	1.6	30.1	4.8	10.6
n-HKUST-1	30.8	6.5	16.2	2.1	27.3	6.6	10.4
s-HKUST-1	33.7	4.3	15.9	1.7	28.2	5.7	10.5

## Data Availability

Data are contained within the article.
